# Complications After Childbirth‐Related Perineal Trauma up to Six‐Weeks Postpartum: A Prospective Cohort Study

**DOI:** 10.1111/1471-0528.18356

**Published:** 2025-09-03

**Authors:** Rebecca Man, R. Katie Morris, Laura Magill, Terry Hughes, Rita Perry, Sue Tohill, Nyesha Henry, Bryar Kadir, Christine Macarthur, Victoria Hodgetts Morton, the CHAPTER group, Laura Jones, John Maltby, Sara Webb, Sarah Hillman, Nicola J. Adderley, Alice Sitch, Olalekan Lee Aiyegbusi, Marian Knight, Krishnarajah Nirantharakumar, Jane Whitehurst, Sophie Webster, Thomas James Curtis, Lily French, Sara Bennett, Laura Johnson, Milli Edwards, Mary Catherine Patterson, Shahla Bakhtiari, Ruth Davies, Abby Rand, Manjula Subramanian, Sana Usman, Katy Wickham, Julie Grindey, Helen Hollins Amphlett, Lindsay E. Bennett, Suneetha Rachaneni, Catherine Bressington, Sujatha Thamban, Stephanie Grigsby, Mollie Jones, Emmanuel I. Emovon, Tessa Dean, Lizzie Phillips, Jenny Butler, Hannah Cook, Lucy Dudgeon, Anna Williams, Hilary Owen, Daisy Elizabeth Pegler, Emily Marler, Amy Mahdi, Vikki Drake

**Affiliations:** ^1^ Department of Applied Health Sciences, School of Health Sciences, College of Medicine and Health University of Birmingham Birmingham UK; ^2^ Birmingham Women's Hospital Birmingham UK

**Keywords:** episiotomy, obstetric anal sphincter injury, perineal tear, postpartum

## Abstract

**Objective:**

To determine the prevalence of perineal wound infection after childbirth‐related perineal trauma up to 6 weeks postpartum.

**Design:**

Prospective, multi‐centre cohort study.

**Setting:**

Fifty‐one UK NHS maternity services.

**Population:**

There were 2021 women who consented to take part and 1213 women who completed their 6‐week questionnaire.

**Methods:**

All women aged 16 or over who sustained childbirth‐related perineal trauma of any type after spontaneous or assisted vaginal birth were eligible. Medical record review and distribution of questionnaires took place at 6 weeks postpartum.

**Main Outcome Measures:**

The primary outcome was perineal wound infection within 6 weeks postpartum, determined using medical record data and questionnaire response.

**Results:**

Six‐week medical record check data was available for 1998 women (99%). 1213 (60%) of women completed their 6‐week questionnaire. 70.6% of consented women experienced a spontaneous vaginal birth and 29.4% of women an assisted vaginal birth. Across all types of perineal trauma and all modes of birth, the overall rate of perineal wound infection was 5.5% (95% CI 4.6%–6.6%). When considering the rate of wound infection by type of tear, women with episiotomy had the highest rate of wound infection at 9.5% (95% CI 7.5%–11.9%).

**Conclusion:**

We provide the best estimate to date of perineal wound infection after childbirth‐related perineal trauma. Improvements to care to reduce perineal wound infection rates are urgently needed, particularly for groups where currently there are no targeted interventions postnatally, such as women who undergo episiotomy after spontaneous vaginal birth.

## 1 Introduction

Childbirth‐related perineal trauma affects the majority of women after vaginal childbirth [1, 2]. Estimates of subsequent perineal wound infection and dehiscence currently range between 0.1% and 23.6% and 0.21% and 24.6% in existing literature [3]. This variation in true incidence is likely due to differences in reporting behaviours, information recording methods and different outcome definitions between studies. Whilst most women are thought not to experience long‐lasting health problems after perineal trauma of any degree, for those that do, the impact can be significant [4].

In addition to the development of perineal wound infection, which can compound or precipitate other more serious complications [5], childbirth‐related perineal trauma can result in several physical and psychological complications, including incontinence, dyspareunia, and post‐traumatic stress symptoms [6, 7, 8, 9, 10]. Estimates on the proportion of women affected by these complications vary between studies. Dyspareunia has been estimated as affecting approximately 70% of women at 1 month after vaginal childbirth [2], with 36% of women who sustain a spontaneous tear affected at 1 year postpartum [9]. In recent Swedish studies, rates of urinary incontinence at 8 weeks postpartum were estimated at between 12.2% and 15.5% after second‐degree perineal trauma and 15.6% and 21.4% after obstetric anal sphincter injury (OASI) [11, 12]. Quantification of the rate of perineal wound infection after perineal trauma is to date limited—retrospective studies using primary and/or secondary care records are unlikely to capture the full scale of the problem. Without accurate and up‐to‐date information on the incidence of these complications after childbirth‐related perineal trauma, targeting resources to avoid, treat, or manage these complications is not possible.

We report the rates of perineal wound infection up to 6 weeks postpartum amongst a diverse group of UK women who have sustained childbirth‐related perineal trauma of any type. Additionally, we report the incidence of broader physical and psychological complications after perineal trauma.

## 2 Materials and Methods

A detailed protocol for the CHAPTER cohort study has been previously published [13]. This was a prospective, multi‐centre cohort study undertaken in the UK. The cohort study follows up women at three time‐points: 6 weeks, 6 months and 12 months postpartum; however, here we present the 6‐week methods and analysis only. This project is funded by the National Institute for Health and Care Research (NIHR), grant number NIHR202869.

### 2.1 Inclusion and Exclusion Criteria

The CHAPTER cohort study was open to any unit in the UK which provided maternity care. This included obstetric‐led and midwifery‐led units, in addition to freestanding midwifery units. In terms of individual inclusion criteria, all women who sustained childbirth‐related perineal trauma were eligible for enrolment into the study. This included women aged 16 or over who underwent spontaneous or assisted vaginal birth with any degree of subsequent perineal trauma, where they were willing and able to give informed consent.

### 2.2 Data Collection and Outcomes

The prospective cohort study initially comprised the collection of baseline clinical information, followed by a medical record check at 6 weeks postpartum, alongside a questionnaire sent to women at the same time point. Baseline information collected included demographic details, information about the current birth, perineal trauma type (first, second, third, or fourth degree tear, episiotomy, or other type of perineal trauma [to include tears not encompassed by the former categories, such as labial tears]), medical conditions, and obstetric history. Medical record checks at 6 weeks postpartum captured complications for which women represented to secondary care, for example through maternity triage pathways. Medical record checks were completed by the research teams at each individual site, using their trust‐specific medical records system. The 6‐week questionnaire sent to women included questions on potential postnatal complications, including perineal wound infection, measures of incontinence, pain, and psychological wellbeing. Through the questionnaire, we aimed to capture potential complications which would not have been recorded via the medical records check. The inclusion of questionnaire data in addition to the medical record check information reduced the risk of underestimating the burden of complications, which would have been the case had there been no patient‐reported components.

The primary outcome was perineal wound infection diagnosed within 6 weeks postpartum. The occurrence of perineal infection was determined using both patient‐reported and clinician‐reported (via the medical record check) components. Perineal wound infection was recorded where women were prescribed antibiotics for a presumed perineal infection, as self‐reported by women or determined from the 6‐week medical records check. Perineal wound infection was also counted where women reported they had been informed by a healthcare professional they had a perineal wound infection.

Secondary outcomes included: use of antibiotics for perineal wound infection and wound breakdown (to include wound separation reported by the woman, wound infection [according to the definition of wound infection above] or wound dehiscence as reported by a healthcare professional). Additionally: urinary incontinence (clinician reported: if the woman had attended maternity outpatient clinic reporting urinary incontinence, patient reported: (i) measured via the Revised Urinary Incontinence Scale and (ii) if the woman reported problems controlling bladder [yes/no answer]) and faecal incontinence (clinician reported: if the woman had attended maternity outpatient clinic reporting faecal incontinence, patient reported: (i) measured via the Revised Faecal Incontinence Scale and (ii) if the woman reported problems controlling flatus/stool [yes/no answer]) [14, 15]. Further secondary outcomes included: maternity triage visit for a perineal trauma related reason, re‐admission for perineal trauma related complications, need for review in a specialist perineal trauma clinic, need for corrective perineal surgery, use of analgesia for perineal pain, impact on caring responsibilities (patient reported: ability to care for baby or older children affected by perineal problems), measures of quality of life (EQ‐5D‐5L tool [EuroQol 5 dimension, 5 level]) [16], and measures of psychological distress (modified City Birth Trauma Scale and components of the Edinburgh Postnatal Depression Scale) [17, 18].

There is currently no core outcome set for childbirth‐related perineal trauma. The outcomes were therefore chosen via consensus with the wider CHAPTER co‐applicants and the Patient Advisory Group (PAG), as detailed in the latter methods section.

### 2.3 Sample Size

The minimum sample size requirement was 1000 women– 300 participants would allow us to determine a complication prevalence rate of 10% with a 95% confidence interval (CI) of 6.8%–14.0%. A sample size of 900 women would enable less common outcomes to be estimated precisely, for example, a 95% CI for an estimate of 5% would be 3.7%–6.6%.

### 2.4 Recruitment

During the course of two defined 14‐day recruitment periods (starting September 2023, ending January 2024), potentially eligible women were identified by the clinical team caring for the woman, or the site research team. After each recruitment period, demographics were reviewed to ensure the study was fully representative of the population, both in terms of ethnicity, location, and type of tear sustained. Women were approached and consented in person after birth or after discharge via telephone—this was to ensure that all women were given the opportunity to take part, including those who were discharged immediately after birth or those who birthed in a non‐hospital setting. Interpreters were used by sites where required. Women were provided with background information on the CHAPTER cohort study, including the study aims and what would be involved if they chose to participate. A written patient information sheet was provided and women had the opportunity to ask any questions that arose. Those who opted to participate gave written informed consent.

### 2.5 Data Management and Analyses

Baseline data was entered by research staff at the site directly on to the RedCap system [19]. Where baseline information data was erroneously inputted by sites (as detected by manually checking for significantly outlying data), and the correction was apparent, then self‐evident corrections were made by the research team. These corrections were discussed and agreed on by consensus of authors and an audit trail was created within the RedCap system. Where the correction was not obvious, then evidently erroneous data was excluded from analysis.

Where women had experienced more than one type of childbirth‐related perineal trauma (e.g., an episiotomy and a third degree tear), all types of perineal trauma experienced were recorded. However, for the purposes of analysis of baseline tear details and outcomes subgrouped by type of tear, the most severe tear was counted only in order to avoid double counting. The ranking of tears was as follows, from least to most severe: other, first‐degree, second‐degree, episiotomy, third‐degree, fourth‐degree.

Proportions and 95% CIs (Wilson method) were calculated for dichotomous outcomes. Where outcomes were continuous, means and standard deviations were calculated. R and Stata software were used.

### 2.6 Sensitivity Analyses

For the primary outcome of perineal wound infection, a sensitivity analysis was performed including only women who responded within the 6 ± 1‐week time window from birth, as this was pre‐specified in our protocol. For all other analyses, all women who responded to their 6‐week questionnaire at any time point were included. We additionally performed sensitivity analyses considering the primary outcome in terms of the rate of patient‐reported wound infection only, the rate of clinician‐reported wound infection only, the rate of clinician‐reported wound infection only from those who completed their 6‐week questionnaire, and the overall rate of wound infection from only those who completed their 6‐week questionnaire.

### 2.7 Patient and Public Involvement

Our PAG guided all aspects of this cohort study, from design of study, including outcomes and mode of questionnaire, through to recruitment, running of the study, and dissemination of results. The PAG were able to guide with interim reviews of the demographics of women included in the study to ensure the study was representative of the UK population. They also provided valuable steer on how to encourage questionnaire uptake throughout the cohort lifespan. At latter study stages, the PAG were also instrumental in the interpretation of results.

### 2.8 Research Inclusion

To facilitate inclusion of women across groups where language barriers existed, interpreters could be used for the consent process. Additionally, questionnaires could be completed via telephone if preferred, enabled by a research midwife and interpreter.

### 2.9 Safety Considerations

Women had the option to request contact from the CHAPTER research midwife if they felt distressed by their experiences or from completing the questionnaire (distress pathway Figure S1). Women could then be appropriately signposted to local services.

## 3 Results

Women were recruited from 51 sites across the United Kingdom (Table S1), in two waves of recruitment between September 2023 and January 2024. There were 4826 women screened across both recruitment waves, of which 4044 were eligible. Of the women screened and subsequently eligible, 2021 women consented to take part in the study, and 6‐week medical record checks were completed for 1998 (99%) of these women. 1213 (60%) women subsequently returned their 6‐week questionnaires. Primary outcome data was available for 2009 (99%) women using both data sources (Figure S2).

Prior to the index birth, 56.7% of women were nulliparous. There were 9.6% of women with any type of diabetes prior to or during pregnancy and 7.2% who were active smokers. 9.5% of women had a body mass index (BMI) ≥ 35 kg/m^2^. Nine women (0.4%) had female genital mutilation identified. Baseline demographic features are presented in Table 1. There were 25 women (2.1%) who activated the distress pathway and requested contact from the study team.

**TABLE 1:** Baseline characteristics.

Characteristic	*N* = 2021, Women consented	*N* = 1213, Returned 6 week questionnaire
Age		
< 18 years	8 (0.4%)	7 (0.6%)
18 to 29 years	865 (42.8%)	483 (39.9%)
30 to 39 years	1076 (53.2%)	684 (56.4%)
40 years or over	72 (3.6%)	39 (3.2%)
BMI		
< 18.50	56 (2.8%)	31 (2.6%)
18.50 to 24.99	841 (42.1%)	527 (43.9%)
25.00 to 34.99	911 (45.6%)	526 (43.8%)
35.00 or over	189 (9.5%)	116 (9.7%)
Missing	24	13
Ethnicity		
Black, Black British, Caribbean or African	94 (4.7%)	47 (3.9%)
Other	135 (6.7%)	73 (6.0%)
Asian or Asian British	297 (14.7%)	136 (11.2%)
White	1491 (73.8%)	955 (78.7%)
Declined to give information	4 (0.2%)	2 (0.2%)

Birth and tear characteristics are available in Tables 2 and 3 respectively. Notably, 70.6% of women in the consented cohort underwent a spontaneous vaginal birth, with the remainder experiencing assisted vaginal birth (12.2% ventouse, 18.3% forceps). The most common type of perineal trauma sustained was a second‐degree tear (38.0%), followed by episiotomy (34.9%). In terms of OASI, 5% of women in the cohort sustained a third‐degree tear and 0.1% of women a fourth‐degree tear. One third of women (33.3%) received antibiotics either during labour or immediately postpartum (therefore not for the treatment of a perineal wound infection).

**TABLE 2:** Labour and birth characteristics.

Characteristic	*N* = 2021, Women consented	*N* = 1213, Returned 6 week questionnaire
Parity prior to index birth		
0	1144 (56.7%)	705 (58.2%)
1	601 (29.8%)	369 (30.5%)
2	184 (9.1%)	103 (8.5%)
≥ 3	88 (4.3%)	34 (2.8%)
Missing	4	2
Onset of labour		
Spontaneous	1109 (55.0%)	669 (55.2%)
Induced	907 (45.0%)	542 (44.8%)
Missing	5	2
Birth type^a^		
Spontaneous birth	1422 (70.6%)	841 (69.6%)
Rotational suction birth	41 (2.0%)	23 (1.9%)
Non‐rotational suction birth	204 (10.2%)	128 (10.6%)
Forceps birth	368 (18.3%)	233 (19.3%)
Gestational week at birth		
< 37 weeks	122 (6.1%)	81 (6.7%)
≥ 37 weeks	1893 (93.9%)	1128 (93.3%)
Missing	6	4
Singleton/twins in this pregnancy		
Singleton	2000 (99.2%)	1200 (99.1%)
Twins	17 (0.8%)	11 (0.9%)
Missing	4	2
Gestational weight		
< 2500 g	114 (5.6%)	66 (5.4%)
2500–3499 g	1157 (57.4%)	686 (56.6%)
3500–4499 g	727 (36.1%)	450 (37.1%)
4500–4999 g	18 (0.9%)	9 (0.7%)
Missing	5	2

^a^ Some women may have had more than one birth mode, for example, a ventouse followed by forceps.

**TABLE 3:** Types of perineal trauma.

Perineal trauma type^a^	*N* = 2021, Women consented	*N* = 1213, Returned 6 week questionnaire
Other lacerations or tears	190 (9.5%)	100 (8.3%)
1st degree	250 (12.5%)	143 (11.9%)
2nd degree	762 (38.0%)	463 (38.4%)
Episiotomy	699 (34.9%)	434 (36.0%)
3a/3b/3c degree tear	100 (5.0%)	63 (5.2%)
4th degree tear	3 (0.1%)	2 (0.2%)
Missing	17	8

^a^ Where a woman had more than one type of perineal trauma, the most severe type is counted in analysis to avoid double counting. For ranking of perineal trauma, see methods.

Across both spontaneous and assisted vaginal births and all perineal trauma types, 5.5% (95% CI 4.6%–6.6%) of women experienced a perineal wound infection within 6 weeks of birth (Table 4). For spontaneous vaginal births and assisted vaginal births, the overall perineal wound infection rates were 4.5% (95% CI 3.5%–5.7%) and 7.9% (95% CI 6.0%–10.4%) respectively. Women who underwent an episiotomy compared to any other tear type had the highest rates of perineal wound infection, with 13.1% (95% CI 9.0%–18.6%) of women with an episiotomy experiencing perineal wound infection after spontaneous vaginal birth and 8.0% (95% CI 5.9%–10.7%) after assisted vaginal birth. Table S2 presents data for the primary outcome of wound infection broken down by clinician reported (6‐week medical record check data) and patient reported (questionnaire), demonstrating rates of 2.5% and 6.8% respectively. In addition, it shows the sensitivity analysis of the primary outcome including only women who completed their questionnaire within 6 ± 1 weeks of birth (8% rate of perineal wound infection).

**TABLE 4:** Perineal infection and perineal wound breakdown outcomes.

Perineal tear type					
Other	First	Second	Episiotomy	Third or fourth degree	Total
*N* (%) CI	*N* (%) CI	*N* (%) CI	*N* (%) CI	*N* (%) CI	*N* (%) CI
*Perineal infection by type of tear and overall, across all modes of birth*					
4 (2.1%), 0.01–0.05	3 (1.2%), 0.00–0.04	29 (3.8%), 0.03–0.05	66 (9.5%), 0.08–0.12	9 (8.7%), 0.05–0.16	111 (5.5%), 0.05–0.07
*Perineal infection by type of tear and overall for spontaneous vaginal births*					
3 (1.7%), 0.01–0.05	3 (1.3%), 0.00–0.04	27 (3.7%), 0.03–0.05	25 (13.1%), 0.09–0.19	5 (8.1%), 0.04–0.18	63 (4.5%), 0.04–0.06
*Perineal infection by type of tear and overall for assisted vaginal births*					
1 (14.3%), 0.03–0.51	0 (0%), 0.00–0.28	2 (6.1%), 0.02–0.20	40 (8.0%), 0.06–0.11	4 (10%), 0.04–0.23	47 (7.9%), 0.06–0.10
*Perineal wound breakdown by type of tear and overall, across all modes of birth* ^a^					
5 (2.6%), 0.01–0.06	8 (3.2%), 0.02–0.06	41 (5.4%), 0.04–0.07	91 (13%), 0.11–0.16	12 (11.7%), 0.07–0.19	158 (7.8%), 0.07–0.09
*Perineal wound breakdown by type of tear and overall for spontaneous vaginal births* ^a^					
3 (1.6%), 0.01–0.05	7 (2.9%), 0.01–0.06	39 (5.4%), 0.04–0.07	32 (16.7%), 0.12–0.23	5 (8.1%), 0.04–0.18	87 (6.1%), 0.05–0.08
*Perineal wound breakdown by type of tear and overall for assisted vaginal births*					
2 (28.6%), 0.08–0.64	1 (10%), 0.02–0.40	2 (5.9%), 0.02–0.19	58 (11.5%), 0.09–0.15	7 (17.5%), 0.09–0.32	70 (11.7%), 0.10–0.15

Abbreviation: CI = confidence interval (95%).

^a^ One patient with wound breakdown had missing perineal trauma type.

Across spontaneous and assisted vaginal births and all perineal trauma types, 7.8% (95% CI 6.7%–9.1%) of women experienced a perineal wound breakdown within 6 weeks of birth, with women in the episiotomy group having the highest rates of wound breakdown (13.0%, 95% CI 10.7%–15.7%) (Table 4). For spontaneous vaginal births and assisted vaginal births, the rates of perineal wound breakdown were 6.1% (95% CI 5.0%–7.5%) and 11.7% (95% CI 9.4%–14.6%) respectively. For women who experienced spontaneous vaginal birth, those who underwent an episiotomy in comparison to any other perineal trauma type had the highest rates of perineal wound breakdown, at 16.7% (95% CI 12.1%–22.6%).

In terms of patient reported outcomes relating to incontinence, 36.5% (95% CI 33.7%–39.4%) of women across all perineal trauma types reported difficulties controlling their bladder within 6 weeks postpartum. 29.1% (95% CI 26.6%–31.9%) of women across all tear types reported difficulty controlling their bowels (to include passing of flatus or stool), with notably 60.7% (95% CI 48.1%–71.9%) of those sustaining OASI reporting this symptom and 34.2% (95% CI 29.7%–38.9%) of those with episiotomy (Table S3). This contrasts to the very small number of women attending maternity outpatients reporting these symptoms within the 6 weeks after birth, with 0.7% (95% CI 0.4%–1.1%) presenting with urinary incontinence and 0.1% (95% CI 0.0%–0.3%) presenting with faecal incontinence, using information from the medical record checks.

Additional secondary outcome results for further complications are available in Tables S4–S12. Within 6 weeks postpartum, 4.4% (95% CI 4.0%–5.0%) of women visited maternity triage for concerns related to their perineal trauma (Table S7). Of women with an episiotomy, 8.2% (95% CI 6.0%–11%) visited triage for a perineal trauma‐related complication, the highest proportion amongst all perineal trauma types.

When considering health‐related quality of life, 17.0% of women reported problems with mobility, 5.7% problems with washing/dressing, 20.2% problems undertaking usual activities (e.g., work or leisure activities), 44.6% pain or discomfort and 36.1% anxiety/depression (Table S10). Analysing components of the modified City Birth Trauma scale [17], 30.4% of women reported recurrent unwanted memories of the birth, 24.3% reported becoming upset when reminded of the birth and 10.1% reported trying to avoid things reminding them of the birth (Table S11).

## 4 Discussion

### 4.1 Main Findings

In this large UK prospective cohort of women who experienced childbirth‐related perineal trauma, the overall rate of perineal wound infection developing within 6 weeks after childbirth was 5.5%. When considering rates of infection across different types of perineal trauma, the rate of infection was notably high amongst women who experienced episiotomy compared to other types of perineal trauma, at 9.5%. Furthermore, amongst women who underwent episiotomy after spontaneous vaginal birth, the rate of perineal wound infection was 13.1%, which is comparatively higher compared to women who underwent episiotomy after assisted vaginal birth (8%).

### 4.2 Strengths and Limitations

A major strength of this study is that it provides data on perineal wound infection from both women's and medical care record sources, not previously undertaken in such a large sample. In capturing patient‐reported outcomes via questionnaire, we ensure that the prevalence of complications post‐childbirth is not under‐counted. We include contemporaneous data from a large and diverse cohort of women, with wide representation from units across the country, resulting in a comprehensive and robust estimate of perineal wound infection. In providing, to our knowledge, the best estimate to date on the burden of perineal wound infection, we provide data fundamental to the design of future research aiming to improve outcomes after perineal trauma. The consideration of the difference in outcomes across women with different perineal trauma types, including women with episiotomy as a distinct group, further strengthens the contribution of this work.

Limitations of this study include that it was conducted only in the UK, providing data on complications perhaps more relevant to a higher‐income country. Our primary outcome was derived using both patient‐reported and clinician‐reported information. Whilst this has been explored fully in sensitivity analyses separating the two reporting pathways, there may therefore be slight underreporting in the overall primary outcome figure where only the clinician‐reported component was available.

In this study we defined perineal wound infection as a prescription of antibiotics for a perineal wound infection, or where women reported they had been informed by a healthcare professional they had a perineal wound infection. This approach provides a real‐life snapshot of current UK practice and estimates the burden of clinically diagnosed wound infection. Our pragmatic definition of perineal wound infection is also in line with the 2019 ANODE trial investigating prophylactic antibiotics for the prevention of maternal infection after assisted vaginal birth [20], where authors defined infection as a new prescription of antibiotics for confirmed or suspected infection. Whilst there is a possibility that this pragmatic definition overcounts the rate of perineal wound infection, in many settings, microbiological confirmation of wound infection may not be possible and therefore our findings are increasingly generalisable [20]. Furthermore, even in settings where appropriate wound swabs are possible, it has been suggested that the initial diagnosis of wound infection should be made in the presence of multiple indicative symptoms and signs, rather than relying on microbial burden, which may not accurately determine clinical wound infection and may result in delay to treatment [21, 22].

### 4.3 Interpretation

The perineal wound infection rate we report is broadly in keeping with smaller studies, such as the 2014 Norwegian prospective cohort study, which investigated rates of infection after episiotomy and found that wound infection occurred in 9.5% of women (17 of 179 women included) [23]. Elsewhere, the 2019 ANODE trial [20], (as described above) reported that approximately 6% of women in the antibiotics arm and 13% of women in the placebo arm subsequently developed a perineal wound infection within 6 weeks after assisted vaginal birth [24].

As episiotomy remains one of the most common surgical procedures performed worldwide, it is imperative to consider the reasons for high infection rates in this group, and consequently to identify potential strategies for reducing morbidity [25]. Episiotomy, in contrast to a spontaneous perineal tear, is a surgical incision performed on skin presumed contaminated by vaginal, bowel, or skin bacteria [26]; therefore theoretically at higher risk of moving bacteria from outward skin to underlying mucosa and muscle tissue with the use of episiotomy scissors. Additionally, as our prospective cohort represents contemporaneous UK practice, the majority of women who experienced OASI perineal trauma received prophylactic antibiotics, which may explain the comparatively lower rate of perineal wound infection in this group [27].

It is notable that rates of infection were higher amongst women who underwent episiotomy for spontaneous vaginal birth, as opposed to for assisted vaginal birth (13.1% and 8.0% respectively). The majority of our cohort would have received prophylactic antibiotics after assisted vaginal birth, as this represents current UK guidelines following the ANODE trial, which may explain some of the difference in wound infection rates [20, 28]. Additional, purely speculative reasons for the varying perineal wound infection rates depending on mode of birth may also include the location of birth and subsequent perineal repair. Assisted vaginal births are more likely to be undertaken in the operating theatre—a controlled environment, where cleaning solution, drapes and sterile gloves are used routinely.

Additionally, we highlight the disparities in complication rates based on medical record checks compared to patient‐reported data. Using only data from secondary care medical records, the rate of perineal wound infection was 2.5%, which compares to a higher figure of 6.8% using questionnaire responses only. This may be because women present to different healthcare professionals in the postpartum period, including general practitioners—in this study, primary care records were not accessed. When considering other secondary outcomes, 36.5% of women reported via questionnaire difficulties controlling their bladder in the 6 weeks following childbirth; however, using the medical records check data alone, only 0.7% of women presented with urinary incontinence. It is likely that the greatest contributor to this is women not presenting with such symptoms [29]. Reasons for this include a documented climate of normalisation regarding incontinence post‐childbirth [30], in addition to the existing barriers in accessing optimal postpartum healthcare [31, 32].

## 5 Conclusion

In this UK, prospective multi‐centre cohort study including over 2000 women, across all types of childbirth‐related perineal trauma, the rate of wound infection within 6 weeks postpartum was 5.5%. Women with an episiotomy after spontaneous vaginal birth had a high rate of perineal wound infection in comparison to women with other perineal trauma types and different modes of birth. Future interventional research, targeted to groups most in need, is urgently required in order to reduce morbidity in women who experience childbirth‐related perineal trauma.

## Author Contributions

R.M.: formal analysis, investigation, methodology, visualisation, writing‐original draft, writing‐review and editing. R.K.M.: conceptualisation, funding acquisition, methodology, investigation, supervision, writing‐review and editing. L.M.: investigation, supervision. T.H.: data curation, investigation. R.P.: project administration. S.T.: investigation, project administration. N.H.: project administration, data curation. B.K.: formal analysis, investigation. C.M.: investigation, supervision, writing‐review and editing. V.H.M.: conceptualisation, funding acquisition, methodology, investigation, supervision, writing‐review and editing.

## Ethics Statement

This study underwent ethical review prior to commencement and was approved by the Research Ethics Council with reference 23/WA/0169 on 27th June 2023. Written informed consent was obtained from all women prior to enrolment into the study. Data was held in accordance with the Data Protection Act (2018 and subsequent amendments) and was registered with the Data Protection Officer at the University of Birmingham.

## Conflicts of Interest

The authors declare no conflicts of interest.

## Supporting information


**Figure S1:** Distress pathway.

**Figure S2:** Study flow diagram.

**Table S1:** Sites from which women took part in the study.
**Table S2:** Perineal wound infection broken down by those reported in medical record checks and those reported in patient questionnaire.
**Table S3:** Difficulty controlling bladder and bowels as per patient reported outcomes.
**Table S4:** Antibiotics given for perineal wound infection within 6 weeks post‐delivery.
**Table S5:** Revised urinary incontinence scale scores (mean and SD).
**Table S6:** Revised faecal incontinence scale (0[no symptoms]‐20[severe]).
**Table S7:** Visits/impact on secondary care as a result of complications after childbirth related perineal trauma within 6 weeks post‐delivery.
**Table S8:** Requirement for analgesia to manage perineal pain at 6 weeks postpartum.
**Table S9:** Impact on caring responsibilities at 6 weeks postpartum.
**Table S10:** Health related quality of life at 6 weeks postpartum‐EQ5D5L responses to each component.
**Table S11:** Patient response to items from the modified City Birth Trauma Scale.
**Table S12:** Patient response to components of the Edinburgh Postnatal Depression Scale.

## Data Availability

The data that support the findings of this study are available from the corresponding author upon reasonable request.
