# Supplementary material for: Complications After Childbirth‐Related Perineal Trauma up to Six‐Weeks Postpartum: A Prospective Cohort Study
Source: BJOG. 2025 Sep 3;133(2):274–82. doi: 10.1111/1471-0528.18356 (PMC12678037; doi:10.1111/1471-0528.18356)
Supplement: Supplementary file 1 — Figure S1: Distress pathway. [file BJO-133-274-s001.pptx]

## Slide 1
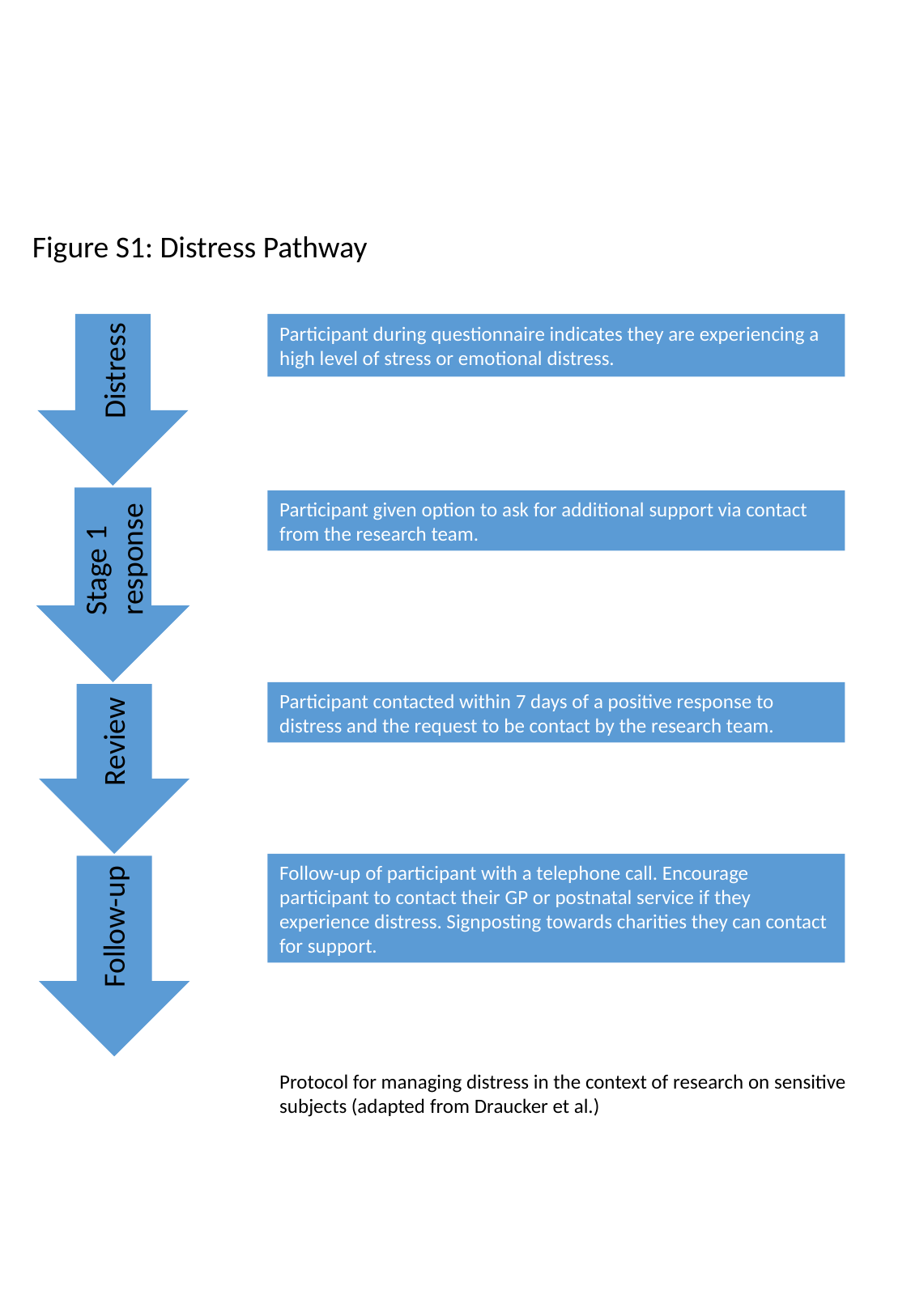

Figure S1: Distress Pathway
Distress
Participant during questionnaire indicates they are experiencing a high level of stress or emotional distress.
Participant given option to ask for additional support via contact from the research team.
Stage 1 response
Participant contacted within 7 days of a positive response to distress and the request to be contact by the research team.
Review
Follow-up of participant with a telephone call. Encourage participant to contact their GP or postnatal service if they experience distress. Signposting towards charities they can contact for support.
Follow-up
Protocol for managing distress in the context of research on sensitive subjects (adapted from Draucker et al.)
