# Supplementary material for: Complications After Childbirth‐Related Perineal Trauma up to Six‐Weeks Postpartum: A Prospective Cohort Study
Source: BJOG. 2025 Sep 3;133(2):274–82. doi: 10.1111/1471-0528.18356 (PMC12678037; doi:10.1111/1471-0528.18356)
Supplement: Supplementary file 2 — Figure S2: Study flow diagram. [file BJO-133-274-s003.pptx]

## Slide 1
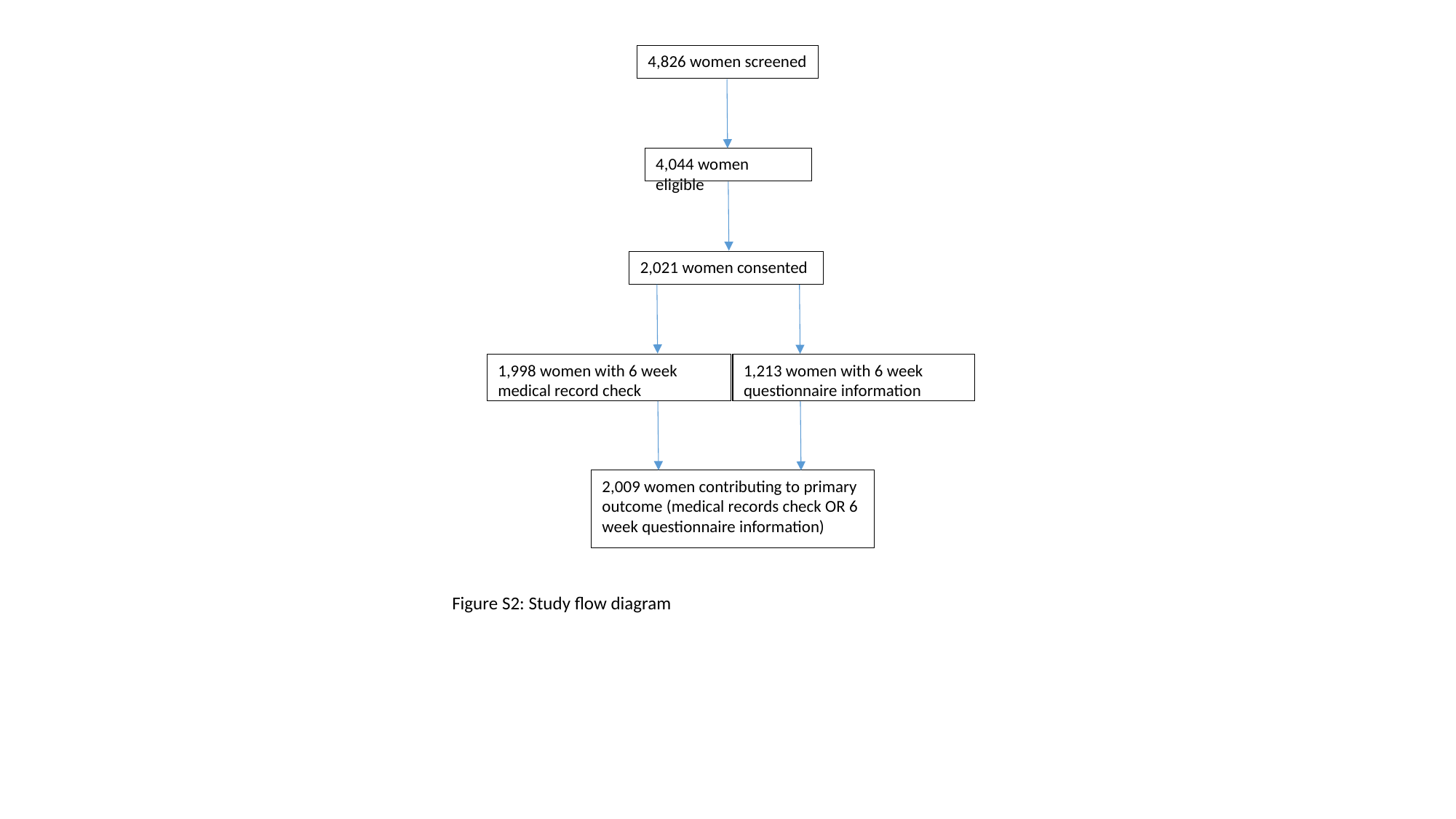

4,826 women screened
4,044 women eligible
2,021 women consented
1,998 women with 6 week medical record check
1,213 women with 6 week questionnaire information
2,009 women contributing to primary outcome (medical records check OR 6 week questionnaire information)
Figure S2: Study flow diagram
