# Supplementary material for: Complications After Childbirth‐Related Perineal Trauma up to Six‐Weeks Postpartum: A Prospective Cohort Study
Source: BJOG. 2025 Sep 3;133(2):274–82. doi: 10.1111/1471-0528.18356 (PMC12678037; doi:10.1111/1471-0528.18356)
Supplement: Supplementary file 3 — Table S1: Sites from which women took part in the study. Table S2: Perineal wound infection broken down by those reported in medical record checks and those reported in patient questionnaire. Table S3: Difficulty controlling bladder and bowels as per patient reported outcomes. Table S4: Antibiotics given for perineal wound infection within 6 weeks post‐delivery. Table S5: Revised urinary incontinence scale scores (mean and SD). Table S6: Revised faecal incontinence scale (0[no symptoms]‐20[severe]). Table S7: Visits/impact on secondary care as a result of complications after childbirth related perineal trauma within 6 weeks post‐delivery. Table S8: Requirement for analgesia to manage perineal pain at 6 weeks postpartum. Table S9: Impact on caring responsibilities at 6 weeks postpartum. Table S10: Health related quality of life at 6 weeks postpartum‐EQ5D5L responses to each component. Table S11: Patient response to items from the modified City Birth Trauma Scale. Table S12: Patient response to components of the Edinburgh Postnatal Depression Scale. [file BJO-133-274-s002.docx]

Table S1: sites from which women took part in the study

| **Unit patient from** | **N= 1,998 (%) Those with completed 6 week medical record checks** | **N= 1,213 (%)**  **Those who returned 6 week questionnaire** |
| --- | --- | --- |
| Arrowe Park Hospital | 37 (1.9) | 21 (1.7) |
| Birmingham City Hospital | 14 (0.7) | 10 (0.8) |
| Birmingham Women’s Hospital | 117 (5.9) | 64 (5.3) |
| Blackpool Victoria Hospital | 40 (2.0) | 24 (2.0) |
| Bradford Royal Infirmary | 35 (1.8) | 17 (1.4) |
| Burnley General Hospital | 70 (3.5) | 49 (4.0) |
| Calderdale Royal Hospital | 62 (3.1) | 41 (3.4) |
| Countess of Chester Hospital | 25 (1.3) | 17 (1.4) |
| Derriford Hospital | 28 (1.4) | 21 (1.7) |
| Doncaster Royal Infirmary | 14 (0.7) | 5 (0.4) |
| Dorset County Hospital | 18 (0.9) | 13 (1.1) |
| George Eliot Hospital | 33 (1.7) | 22 (1.8) |
| Great Western Hospital | 44 (2.2) | 32 (2.6) |
| Heartlands Hospital Birmingham | 25 (1.3) | 15 (1.2) |
| Hereford County Hospital | 9 (0.5) | 8 (0.7) |
| Hillingdon Hospital | 68 (3.4) | 24 (2.0) |
| Kings College Hospital | 76 (3.8) | 51 (4.2) |
| Kingston Hospital | 68 (3.4) | 45 (3.7) |
| Leicester Royal Infirmary | 59 (3.0) | 31 (2.6) |
| Leighton Hospital | 18 (0.9) | 12 (1.0) |
| Liverpool Women’s Hospital | 25 (1.3) | 11 (0.9) |
| Musgrove Park Hospital | 38 (1.9) | 23 (1.9) |
| Norfolk and Norwich University Hospitals NHS Foundation Trust | 40 (2.0) | 26 (2.1) |
| Northampton General Hospital | 28 (1.4) | 11 (0.9) |
| Northumbria Specialist Emergency Care Hospital | 20 (1.0) | 14 (1.2) |
| Ormskirk District General Hospital | 35 (1.8) | 24 (2.0) |
| Poole Hospital | 93 (4.7) | 63 (5.2) |
| Princess of Wales Hospital, Glamorgan | 19 (1.0) | 16 (1.3) |
| Princess Royal Hospital, Haywards Heath | 16 (0.8) | 13 (1.1) |
| Princess Royal Hospital, Telford | 20 (1.0) | 9 (0.7) |
| Queen Elizabeth Hospital Gateshead | 28 (1.4) | 25 (2.1) |
| Queens Hospital Burton | 23 (1.2) | 17 (1.4) |
| Rosie Hospital, Cambridge | 110 (5.5) | 73 (6.0) |
| Royal Bolton Hospital | 103 (5.2) | 59 (4.9) |
| Royal Derby Hospital | 45 (2.3) | 26 (2.1) |
| Royal Devon and Exeter Hospital | 16 (0.8) | 9 (0.7) |
| Royal Free Hospital | 37 (1.9) | 16 (1.3) |
| Royal Gwent Hospital | 37 (1.9) | 22 (1.8) |
| Royal Sussex County Hospital | 24 (1.2) | 17 (1.4) |
| Royal United Hospital, Bath | 42 (2.1) | 24 (2.0) |
| Russells Hall Hospital, Dudley | 23 (1.2) | 13 (1.1) |
| Salisbury District Hospital | 38 (1.9) | 26 (2.1) |
| Scunthorpe General Hospital | 23 (1.2) | 19 (1.6) |
| Stepping Hill Hospital | 29 (1.5) | 22 (1.8) |
| The Royal London Hospital | 25 (1.3) | 9 (0.7) |
| Tunbridge Wells Hospital | 47 (2.4) | 37 (3.1) |
| University Hospital Lewisham | 37 (1.9) | 24 (2.0) |
| Walsall Manor Hospital | 39 (2.0) | 17 (1.4) |
| Whipps Cross University Hospital | 29 (1.5) | 5 (0.4) |
| Glan Clwyd Hospital | 29 (1.5) | 9 (0.7) |
| Ysbyty Gwynedd Hospital | 20 (1.0) | 12 (1.0) |

Table S2: Perineal wound infection broken down by those reported in medical record checks and those reported in patient questionnaire. Sensitivity analysis only including those who returned their questionnaire within the 6+/-1 week window. CI=confidence interval (95%)

| **N = 1,998**  **Clinician reported cases of infection only** | **N=1,213**  **Patient reported cases of infection only** | **N=1,213 Reported in medical record check *only* from those who completed 6 week questionnaire** | **N=1,213 *Overall rate* of infection from those who completed 6 week questionnaire** | **N=842**  **Reported in patient questionnaire─ only including those who returned questionnaire within 6+- 1 week window** |
| --- | --- | --- | --- | --- |
| **N (%) CI** | **N (%)** **CI** | **N (%) CI** | **N (%) CI** | **N (%)** **CI** |
| 50 (2.5%), 0.02-0.03 | 82 (6.8%), 0.05-0.08 | 25 (2.1%), 0.01-0.03 | 86 (7.1%), 0.06-0.09 | 67 (8.0%), 0.06-0.10 |

Table S3: Difficulty controlling bladder and bowels as per patient reported outcomes. CI=confidence interval (95%)

| Other | First | Second | Episiotomy | Third or fourth | **Total^1^** | |
| --- | --- | --- | --- | --- | --- | --- |
| **N (%) CI** | **N (%) CI** | **N (%) CI** | **N (%) CI** | **N (%) CI** | | **N (%) CI** |
| **Patient reported: problems controlling bladder** | | | | | | |
| 28 (32.6%), 0.24-0.43 | 49 (36.8%), 0.29-0.45 | 145 (34%), 0.30-0.39 | 156 (38.9%), 0.34-0.44 | 25 (41.7%), 0.30-0.54 | | **406 (36.5%), 0.34-0.39** |
| **Patient reported: problems controlling bowels (to include flatus/stool)** | | | | | | |
| 16 (18.4%), 0.12-0.28 | 31 (23.1%), 0.17-0.31 | 102 (23.8%), 0.2-0.28 | 138 (34.2%), 0.30-0.39 | 37 (60.7%), 0.48-0.72 | | **326 (29.1%), 0.27-0.32** |

^1^Three women who reported problems controlling their bladder and two women who reported problems controlling flatus/stool had perineal trauma type information missing.

Table S4: Antibiotics given for perineal wound infection within six weeks post-delivery. CI=confidence interval (95%)

| Other | First | Second | Episiotomy | Third or fourth | **Total** |
| --- | --- | --- | --- | --- | --- |
| **N (%) CI** | **N (%) CI** | **N (%) CI** | **N (%) CI** | **N (%) CI** | **N (%) CI** |
| 4 (2.1%), 0.01-0.05 | 2 (0.8%), 0.00-0.03 | 20 (2.6%), 0.02- 0.04 | 60 (8.6%), 0.07-0.11 | 8 (7.8%), 0.04-0.15 | **94 (4.7%), 0.04-0.06** |

Table S5: Revised urinary incontinence scale scores (mean and SD). 0 = no urinary incontinence, 16 = severe urinary incontinence. CI=confidence interval (95%). SD=standard deviation. *The questions in the revised urinary incontinence scale were only asked if participants reported problems controlling their bladder in the initial screening question.*

| Other | | First | | Second | | Episiotomy | | Third or fourth | | **Total** | |
| --- | --- | --- | --- | --- | --- | --- | --- | --- | --- | --- | --- |
| **N** | **Mean (SD) CI** | **N** | **Mean (SD) CI** | **N** | **Mean (SD) CI** | **N** | **Mean (SD) CI** | **N** | **Mean (SD) CI** | **N** | **Mean (SD) CI** |
| 19 | 8.74 (3.62), 7.15-10.32 | 35 | 7.97 (2.76), 7.07-8.87 | 103 | 7.37 (2.89), 6.81-7.93 | 101 | 8.13 (3.27), 7.49-8.76 | 19 | 7.95 (3.39), 6.46-9.43 | **277** | **7.87 (3.11), 7.51-8.24** |

Table S6: Revised faecal incontinence scale (0[no symptoms]-20[severe]). <4 = no faecal incontinence/very mild symptoms, 4-6 = mild, 7-12 = moderate, 13 or above = severe. CI=confidence interval (95%). *The questions in the revised faecal incontinence scale were only asked if participants reported problems controlling their bowels in the initial screening question.*

| Level | Other | First | Second | Episiotomy | Third or fourth | **Total** |
| --- | --- | --- | --- | --- | --- | --- |
|  | **N (%) CI** | **N (%) CI** | **N (%) CI** | **N (%) CI** | **N (%) CI** | **N (%) CI** |
| Score < 4 | 9 (60.0%), 0.357-0.802 | 21 (70.0%), 0.521-0.833 | 66 (67.3%), 0.576- 0.758 | 69 (53.9%), 0.453-0.623 | 14 (38.9%), 0.248- 0.551 | **179 (58.3%), 0.527- 0.636** |
| Score 4 - 6 | 2 (13.3%), 0.037-0.379 | 2 (6.7%), 0.018-0.213 | 18 (18.4%), 0.119- 0.272 | 27 (21.1%), 0.149-0.29 | 8 (22.2%), 0.117- 0.381 | **56 (18.4%), 0.145- 0.231** |
| Score 7 - 12 | 2 (13.3%), 0.037-0.379 | 4 (13.3%), 0.053-0.297 | 12 (12.2%), 0.071- 0.202 | 19 (14.8%), 0.097-0.22 | 9 (25.0%), 0.138-0.411 | **47 (15.2%), 0.116- 0.196** |
| Score ≥13 | 2 (13.3%), 0.037- 0.379 | 3 (10.0%), 0.035-0.256 | 2 (2.0%), 0.006- 0.071 | 13 (10.2%), 0.06-0.166 | 5 (13.9%), 0.061- 0.287 | **25 (8.1%), 0.055-0.117** |

Table S7: Visits/impact on secondary care as a result of complications after childbirth related perineal trauma within six weeks post-delivery. CI=confidence interval (95%).

| Other | First | Second | Episiotomy | Third or fourth | **Total** |
| --- | --- | --- | --- | --- | --- |
| **N (%) CI** | **N (%) CI** | **N (%) CI** | **N (%) CI** | **N (%) CI** | **N (%) CI** |
| **Triage visit for CRPT related complications^1^** | | | | | |
| 2 (1.1%), 0.00-0.04 | 3 (1.2%), 0.00- 0.04 | 20 (2.6%), 0.02- 0.04 | 57 (8.2%), 0.06-0.11 | 4 (4%), 0.02-0.10 | **87 (4.4%), 0.04-0.05** |
| **Re-admission for CRPT related complications** | | | | | |
| 0 (0%), 0.00-0.02 | 1 (0.4%), 0.00- 0.02 | 1 (0.1%), 0.00, 0.01 | 7 (1%), 0.01-0.02 | 0 (0%), 0.00-0.04 | **9 (0.5%), 0.00-0.01** |
| **Requiring review at specialist perineal clinic** | | | | | |
| 0 (0%), 0.00-0.02 | 0 (0%), 0-0.02 | 0 (0%), 0.00, 0.01 | 0 (0%), 0.00-0.01 | 0 (0%), 0.00-0.04 | **0 (0%), 0.00-0.00** |
| **Requiring minor or major corrective perineal surgery (after initial repair)** | | | | | |
| 0 (0%), 0.00-0.02 | 0 (0%), 0.00-0.02 | 0 (0%), 0.00, 0.01 | 1 (0.1%), 0.00-0.01 | 0 (0%), 0.00-0.04 | **1 (0.1%), 0.00-0.01** |

^1^One woman who visited triage with a perineal trauma complication had missing perineal trauma type

Table S8: requirement for analgesia to manage perineal pain at six weeks postpartum. CI=confidence interval (95%). *The question regarding analgesia was only asked if participants reported pain in the initial screening question.*

| Other | First | Second | Episiotomy | Third or fourth | **Total** |
| --- | --- | --- | --- | --- | --- |
| **N (%) CI** | **N (%) CI** | **N (%) CI** | **N (%) CI** | **N (%) CI** | **N (%) CI** |
| 5 (29.4%), 0.13-0.53 | 4 (23.5%), 0.1-0.47 | 18 (20.2%), 0.13-0.30 | 44 (35.2%), 0.27-0.44 | 10 (37%), 0.22-0.56 | **81 (29.5%), 0.24-0.35** |

Table S9: impact on caring responsibilities at six weeks postpartum. CI=confidence interval (95%).

| Other | First | Second | Episiotomy | Third or fourth | **Total** |
| --- | --- | --- | --- | --- | --- |
| **N (%) CI** | **N (%) CI** | **N (%) CI** | **N (%) CI** | **N (%) CI** | **N (%) CI** |
| **Breastfeeding stopped as perineum uncomfortable? (Answered yes)** | | | | | |
| 1 (1.4%), 0.00-0.08) | 3 (2.8%), 0.01-0.08 | 3 (0.9%), 0.00-0.03 | 13 (3.9%), 0.02-0.07 | 0 (0%), 0.00-0.07 | **20 (2.2%), 0.01-0.03** |
| **Have problems or worries about your perineum caused you to not be able to care for your older children in the way you would like? (Answered yes)** | | | | | |
| 3 (5.5%), 0.02-0.15 | 7 (6.7%), 0.03-0.13 | 32 (12.5%), 0.09-0.17 | 27 (17.8%), 0.13-0.25 | 5 (16.7%), 0.07-0.34 | **74 (12.3%), 0.10-0.15** |

Table S10: Health related quality of life at six weeks postpartum─ EQ5D5L responses to each component

| **Characteristic** | Other | First | Second | Episiotomy | Third or fourth | **Overall** |
| --- | --- | --- | --- | --- | --- | --- |
| **Mobility** | | | | | | |
| I have no problems in walking about | 74 (89.2%) | 116 (87.9%) | 370 (86.9%) | 312 (78.0%) | 43 (70.5%) | **915 (83.0%)** |
| I have slight problems in walking about | 7 (8.4%) | 9 (6.8%) | 42 (9.9%) | 71 (17.8%) | 15 (24.6%) | **144 (13.1%)** |
| I have moderate problems in walking about | 2 (2.4%) | 6 (4.5%) | 10 (2.3%) | 15 (3.8%) | 1 (1.6%) | **34 (3.1%)** |
| I have severe problems in walking about | 0 (0.0%) | 1 (0.8%) | 2 (0.5%) | 2 (0.5%) | 2 (3.3%) | **7 (0.6%)** |
| I am unable to walk about | 0 (0.0%) | 0 (0.0%) | 2 (0.5%) | 0 (0.0%) | 0 (0.0%) | **2 (0.2%)** |
| **Self-care** | | | | | | |
| I have no problems washing or dressing myself | 79 (96.3%) | 123 (93.9%) | 408 (97.1%) | 366 (91.3%) | 57 (93.4%) | **1,033 (94.3%)** |
| I have slight problems washing or dressing myself | 3 (3.7%) | 7 (5.3%) | 7 (1.7%) | 30 (7.5%) | 4 (6.6%) | **51 (4.7%)** |
| I have moderate problems washing or dressing myself | 0 (0.0%) | 1 (0.8%) | 5 (1.2%) | 5 (1.2%) | 0 (0.0%) | **11 (1.0%)** |
| I have severe problems washing or dressing myself | 0 (0.0%) | 0 (0.0%) | 0 (0.0%) | 0 (0.0%) | 0 (0.0%) | **0 (0.0%)** |
| I am unable to wash or dress myself | 0 (0.0%) | 0 (0.0%) | 0 (0.0%) | 0 (0.0%) | 0 (0.0%) | **0 (0.0%)** |
| **Usual activities (e.g. work, study, housework, family or leisure activities)** | | | | | | |
| I have no problems doing my usual activities | 71 (88.8%) | 111 (83.5%) | 356 (83.8%) | 291 (73.5%) | 44 (73.3%) | **873 (79.8%)** |
| I have slight problems doing my usual activities | 8 (10.0%) | 16 (12.0%) | 57 (13.4%) | 85 (21.5%) | 12 (20.0%) | **178 (16.3%)** |
| I have moderate problems doing my usual activities | 1 (1.3%) | 5 (3.8%) | 11 (2.6%) | 16 (4.0%) | 3 (5.0%) | **36 (3.3%)** |
| I have severe problems doing my usual activities | 0 (0.0%) | 1 (0.8%) | 1 (0.2%) | 3 (0.8%) | 1 (1.7%) | **6 (0.5%)** |
| I am unable to do my usual activities | 0 (0.0%) | 0 (0.0%) | 0 (0.0%) | 1 (0.3%) | 0 (0.0%) | **1 (0.1%)** |
| **Pain/discomfort** | | | | | | |
| I have no pain or discomfort | 53 (64.6%) | 90 (67.7%) | 263 (62.8%) | 173 (43.3%) | 28 (45.9%) | **607 (55.4%)** |
| I have slight pain or discomfort | 23 (28.0%) | 35 (26.3%) | 138 (32.9%) | 181 (45.3%) | 24 (39.3%) | **401 (36.6%)** |
| I have moderate pain or discomfort | 5 (6.1%) | 7 (5.3%) | 13 (3.1%) | 41 (10.3%) | 6 (9.8%) | **72 (6.6%)** |
| I have severe pain or discomfort | 0 (0.0%) | 1 (0.8%) | 4 (1.0%) | 4 (1.0%) | 3 (4.9%) | **12 (1.1%)** |
| I have extreme pain or discomfort | 1 (1.2%) | 0 (0.0%) | 1 (0.2%) | 1 (0.3%) | 0 (0.0%) | **3 (0.3%)** |
| **Anxiety or depression** | | | | | | |
| I am not anxious or depressed | 50 (59.5%) | 88 (66.7%) | 294 (69.2%) | 241 (60.6%) | 32 (54.2%) | **705 (64.2%)** |
| I am slightly anxious or depressed | 20 (23.8%) | 29 (22.0%) | 93 (21.9%) | 104 (26.1%) | 20 (33.9%) | **266 (24.2%)** |
| I am moderately anxious or depressed | 8 (9.5%) | 13 (9.8%) | 32 (7.5%) | 48 (12.1%) | 6 (10.2%) | **107 (9.7%)** |
| I am severely anxious or depressed | 3 (3.6%) | 2 (1.5%) | 3 (0.7%) | 2 (0.5%) | 1 (1.7%) | **11 (1.0%)** |
| I am extremely anxious or depressed | 3 (3.6%) | 0 (0.0%) | 3 (0.7%) | 3 (0.8%) | 0 (0.0%) | **9 (0.8%)** |

Table S11: patient response to items from the modified City Birth Trauma Scale

| **Component, symptom frequency** | Other | First | Second | Episiotomy | Third or fourth | **Overall** |
| --- | --- | --- | --- | --- | --- | --- |
| **Recurrent unwanted memories of the birth (or parts of the birth) that you can’t control** | | | | | | |
| Not at all | 61 (75.3%) | 98 (74.2%) | 336 (79.8%) | 235 (59.2%) | 30 (49.2%) | **760 (69.6%)** |
| Once | 9 (11.1%) | 15 (11.4%) | 33 (7.8%) | 63 (15.9%) | 9 (14.8%) | **129 (11.8%)** |
| 2-4 times | 10 (12.3%) | 14 (10.6%) | 41 (9.7%) | 67 (16.9%) | 18 (29.5%) | **150 (13.7%)** |
| 5 or more times | 1 (1.2%) | 5 (3.8%) | 11 (2.6%) | 32 (8.1%) | 4 (6.6%) | **53 (4.9%)** |
| **Bad dreams or nightmares about the birth (or related to the birth)** | | | | | | |
| Not at all | 74 (90.2%) | 121 (91.7%) | 396 (94.5%) | 334 (84.8%) | 54 (88.5%) | **979 (90.0%)** |
| Once | 5 (6.1%) | 6 (4.5%) | 15 (3.6%) | 32 (8.1%) | 5 (8.2%) | **63 (5.8%)** |
| 2-4 times | 1 (1.2%) | 3 (2.3%) | 7 (1.7%) | 22 (5.6%) | 2 (3.3%) | **35 (3.2%)** |
| 5 or more times | 2 (2.4%) | 2 (1.5%) | 1 (0.2%) | 6 (1.5%) | 0 (0.0%) | **11 (1.0%)** |
| **Flashbacks to the birth and/or reliving the experience** | | | | | | |
| Not at all | 54 (68.4%) | 89 (68.5%) | 287 (68.3%) | 211 (53.7%) | 32 (52.5%) | **673 (62.1%)** |
| Once | 12 (15.2%) | 19 (14.6%) | 42 (10.0%) | 57 (14.5%) | 12 (19.7%) | **142 (13.1%)** |
| 2-4 times | 10 (12.7%) | 16 (12.3%) | 63 (15.0%) | 78 (19.8%) | 12 (19.7%) | **179 (16.5%)** |
| 5 or more times | 3 (3.8%) | 6 (4.6%) | 28 (6.7%) | 47 (12.0%) | 5 (8.2%) | **89 (8.2%)** |
| **Getting upset when reminded of the birth** | | | | | | |
| Not at all | 63 (76.8%) | 112 (84.2%) | 361 (85.1%) | 256 (65.0%) | 36 (59.0%) | **828 (75.7%)** |
| Once | 12 (14.6%) | 9 (6.8%) | 35 (8.3%) | 58 (14.7%) | 9 (14.8%) | **123 (11.2%)** |
| 2-4 times | 6 (7.3%) | 10 (7.5%) | 18 (4.2%) | 52 (13.2%) | 8 (13.1%) | **94 (8.6%)** |
| 5 or more times | 1 (1.2%) | 2 (1.5%) | 10 (2.4%) | 28 (7.1%) | 8 (13.1%) | **49 (4.5%)** |
| **Feeling tense or anxious when reminded of the birth** | | | | | | |
| Not at all | 65 (80.2%) | 103 (78.0%) | 354 (83.5%) | 259 (65.2%) | 34 (56.7%) | **815 (74.5%)** |
| Once | 9 (11.1%) | 17 (12.9%) | 34 (8.0%) | 59 (14.9%) | 13 (21.7%) | **132 (12.1%)** |
| 2-4 times | 4 (4.9%) | 12 (9.1%) | 29 (6.8%) | 54 (13.6%) | 6 (10.0%) | **105 (9.6%)** |
| 5 or more times | 3 (3.7%) | 0 (0.0%) | 7 (1.7%) | 25 (6.3%) | 7 (11.7%) | **42 (3.8%)** |
| **Trying to avoid things that remind me of the birth** | | | | | | |
| Not at all | 73 (91.3%) | 120 (90.2%) | 404 (95.7%) | 329 (84.1%) | 51 (85.0%) | **977 (90.0%)** |
| Once | 7 (8.8%) | 5 (3.8%) | 9 (2.1%) | 26 (6.6%) | 5 (8.3%) | **52 (4.8%)** |
| 2-4 times | 0 (0.0%) | 7 (5.3%) | 6 (1.4%) | 25 (6.4%) | 1 (1.7%) | **39 (3.6%)** |
| 5 or more times | 0 (0.0%) | 1 (0.8%) | 3 (0.7%) | 11 (2.8%) | 3 (5.0%) | **18 (1.7%)** |
| **Not able to remember details of the birth** | | | | | | |
| Not at all | 64 (78.0%) | 115 (87.1%) | 348 (82.3%) | 249 (62.9%) | 37 (61.7%) | **813 (74.4%)** |
| Once | 10 (12.2%) | 9 (6.8%) | 31 (7.3%) | 51 (12.9%) | 7 (11.7%) | **108 (9.9%)** |
| 2-4 times | 6 (7.3%) | 6 (4.5%) | 34 (8.0%) | 64 (16.2%) | 10 (16.7%) | **120 (11.0%)** |
| 5 or more times | 2 (2.4%) | 2 (1.5%) | 10 (2.4%) | 32 (8.1%) | 6 (10.0%) | **52 (4.8%)** |
| **Blaming myself or others for what happened during the birth** | | | | | | |
| Not at all | 69 (87.3%) | 109 (83.2%) | 377 (89.1%) | 282 (71.2%) | 40 (65.6%) | **877 (80.5%)** |
| Once | 2 (2.5%) | 13 (9.9%) | 23 (5.4%) | 41 (10.4%) | 7 (11.5%) | **86 (7.9%)** |
| 2-4 times | 8 (10.1%) | 7 (5.3%) | 18 (4.3%) | 47 (11.9%) | 9 (14.8%) | **89 (8.2%)** |
| 5 or more times | 0 (0.0%) | 2 (1.5%) | 5 (1.2%) | 26 (6.6%) | 5 (8.2%) | **38 (3.5%)** |
| **Feeling strong negative emotions about the birth** | | | | | | |
| Not at all | 68 (84.0%) | 103 (79.8%) | 370 (87.3%) | 264 (66.5%) | 39 (63.9%) | **844 (77.3%)** |
| Once | 4 (4.9%) | 17 (13.2%) | 29 (6.8%) | 60 (15.1%) | 6 (9.8%) | **116 (10.6%)** |
| 2-4 times | 5 (6.2%) | 8 (6.2%) | 18 (4.2%) | 44 (11.1%) | 10 (16.4%) | **85 (7.8%)** |
| 5 or more times | 4 (4.9%) | 1 (0.8%) | 7 (1.7%) | 29 (7.3%) | 6 (9.8%) | **47 (4.3%)** |

Table S12: Patient response to components of the Edinburgh Postnatal Depression Scale

| **Characteristic** | **N (%)** |
| --- | --- |
| **I have been able to laugh and see the funny side of things** | |
| As much as I always could | 818 (74.2%) |
| Not quite so much now | 246 (22.3%) |
| Definitely not so much now | 31 (2.8%) |
| Not at all | 7 (0.6%) |
| **I have looked forward with enjoyment to things** | |
| As much as I ever did | 781 (71.1%) |
| Rather less than I used to | 273 (24.8%) |
| Definitely less than I used to | 37 (3.4%) |
| Hardly at all | 8 (0.7%) |
| **I have blamed myself unnecessarily when things went wrong** | |
| No never | 183 (16.6%) |
| Not very often | 350 (31.7%) |
| Yes, some of the time | 456 (41.3%) |
| Yes, most of the time | 116 (10.5%) |
| **I have been anxious or worried for no good reason** | |
| No not at all | 270 (24.5%) |
| Hardly ever | 293 (26.6%) |
| Yes, sometimes | 466 (42.3%) |
| Yes, very often | 72 (6.5%) |
| **I have felt scared or panicky for no very good reason** | |
| No, not at all | 448 (40.9%) |
| No, not much | 301 (27.5%) |
| Yes, sometimes | 299 (27.3%) |
| Yes, quite a lot | 48 (4.4%) |
| **Things have been getting on top of me** | |
| No, I have been coping as well as ever | 197 (17.9%) |
| No, most of the time I have coped quite well | 544 (49.3%) |
| Yes, sometimes I haven’t been coping as well as usual | 336 (30.5%) |
| Yes, most of the time I haven’t been able to cope at all | 26 (2.4%) |
